# Administration of *Streptococcus bovis* isolated from sheep rumen digesta on rumen function and physiology as evaluated in a rumen simulation technique system

**DOI:** 10.14202/vetworld.2019.1362-1371

**Published:** 2019-09

**Authors:** Durgadevi Aphale, Aamod Natu, Sharad Laldas, Aarohi Kulkarni

**Affiliations:** 1 Praj Matrix, Research and Development Center, Division of Praj Industries Ltd., 402/403/1098, Pune, Maharashtra, India; 2 Department of Health and Biological Sciences, Symbiosis International (Deemed University), Pune, Maharashtra, India

**Keywords:** bacteriocin, probiotic, rumen simulation technique, rumen, *Streptococcus bovis*

## Abstract

**Background and Aim:**

Little information about the stability and changes of sheep ruminal microbiota due to pathogen intervention in the rumen simulation technique (RUSITEC) is available. This study aimed to investigate the effect of administration of a novel isolated *Streptococcus bovis* strain on rumen microbiology and physiology. In addition, the isolation of pigment-producing *Streptococcus lutetiensis* is described.

**Materials and Methods:**

Microbial strains were isolated from sheep rumen digesta. An isolated strain of *S. bovis* was evaluated in the RUSITEC system fed with mixed cattle feed and compared with an in-house developed probiotic formulation (PF), PF 1, containing *Bacillus amyloliquifaciens*, *Bacillus subtilis*, and *Propionibacterium freudenreichii*. The parameters of volatile fatty acid, lactic acid, pH profiling, and the coliform anti-pathogenicity were evaluated to determine the effect of *S. bovis* on rumen function and physiology.

**Results:**

Administration of *S. bovis* reduced the coliform count by 31.20% from 7.2×10^10^ colony-forming units (CFU)/mLto 1.7×10^6^ CFU/mL. Agar diffusion assays revealed the extracellular antimicrobial activity of *S. bovis* against coliforms; *Escherichia coli* and *Salmonella enterica* with 12 and 14 mm zones of inhibition, respectively. Simultaneously, an increase of 61.62% in the rumen yeast count was noted. The physiological changes resulted in a 5% reduction in acetic acid concentration from 431 to 405 mg/L.

**Conclusion:**

The present research indicates that *S. bovis* is highly capable of altering rumen physiology and function on colonization and is a key transition microbe to be studied during rumen intervention studies. A decrease in the coliform count could be attributed to extracellular production of a bacteriocin-like substance, as illustrated through agar diffusion assays.

## Introduction

The ruminal microbial community is diverse and is comprised hundreds of different bacterial, archaeal, fungal, and protozoal species. The core microbiome of the rumen is dominated by the phyla *Bacteroidetes* and *Firmicutes*, in addition to many other taxa [1]. Ruminal streptococci represent facultative anaerobic bacteria which are regularly isolated from rumen of cattle and sheep [2], indicating their dominance over other culturable lactic acid-producing bacteria.

Among streptococci, a *Streptococcus bovis* is a facultative anaerobe that is normally found in the rumen of cattle and the colon of monogastrics. Rumen acidosis is associated with an initial overgrowth of *S. bovis* and the inability of lactic acid utilizing bacteria like *Megasphaera elsdenii* to utilize these acids and grow [3]. *S. bovis* produces lactic acid mainly when the pH is lower than 5.5; however, it shifts to formate, acetate, and ethanol fermentation when the pH is higher than 6.0 [4]. It seems that both pH and the fermentation substrate abundance play a critical role in regulating the lactic acid production of *S. bovis*. A drop in ruminal pH often depresses fiber digestion, because major cellulolytic ruminal bacteria are sensitive to low pH (pH <6.0) [5]. Since the rumen microbiome displays fluctuations in response to external interventions, it is considered an ideal environment for microbial ecology research and assertion of ecological principles. However, the microbiome demonstrates redundancy and resilience, limiting the potential of rumen engineering for improved functions. The intense competition and amensalism from the native rumen residents, well-adapted to the prior conditions, hinders the establishment of a new microbial community [1]. *In vitro* systems are most suitable due to these constraints and for the preliminary evaluation of probiotic or pathogen effects on ruminal fermentation and microbial populations. One commonly used *in vitro* system is the semi-continuous rumen simulation technique (RUSITEC), recently used by Wetzels *et al*. [6] for studying the dynamics of the bacterial community during challenge with *Clostridium perfringens*. Isolation of *S. bovis* has been reported from the rumen of dromedary camel, Rusa deer, and bovine rumen [7,8] with rumen fermentation and feed digestion studies [9]. In addition, Joachimsthal *et al*. [10] reported bovicin production from *S. bovis* strains isolated from Australian ruminants. In the present study, *in vitro* evaluation of rumen ecosystem during *S. bovis* intervention was conducted to explain microbial physiology that finally results in an alteration of rumen function.

This study aimed to isolate and preliminarily characterize *Streptococcus* spp. from sheep rumen digesta, followed by *in vitro* evaluation of *S. bovis* intervention on rumen fermentation and microbial population using a RUSITEC system.

## Materials and Methods

### Ethical approval

Ethical approval is not required due to the absence of animal trials.

### Sample collection and processing

All media and chemicals were obtained from HiMedia, India, unless otherwise stated. Isolation of rumen bacteria was conducted using sheep rumen digesta obtained from a government approved slaughterhouse near Pune, Maharashtra, India, under controlled environmental conditions. Rumen content was filtered, based on their nutritional requirements [11]. For filtration, the sheep rumen contents were washed with artificial saliva at a 1:1 ratio. The saliva solution was comprised NaHCO_3_, 9.80 g/L; Na_2_HPO_4_, 4.97 g/L; KCl, 0.57 g/L; NaCl, 0.47 g/L; MgCl_2_, 0.123 g/L; and CaCl_2_, 0.04 g/L and heated to 39±2°C before processing. The resulting solution was gauze filtered.

The unfiltered (2 g) and filtered (2 mL) rumen contents were transported to an anaerobic glove box for microbial isolation and added to 20 mL emulsifier solution containing tween 80 (0.1 g/L, pre-sterilized at 121°C for 20 min)+saline (9 g/L, pre-sterilized, at 121°C for 20 min) that was degassed with N_2_ (1 min). The content was homogenized using a vortex mixer for 10 min.

### Isolation of rumen *Streptococcus* species

Unfiltered and filtered rumen contents were diluted from 10^1^ to 10^10^ using the emulsifier solution. The diluted mixture was spread plated onto different media plates as described below. The plates were placed in the anaerobic glove box for 48 h before use to have an anaerobic environment and then incubated after spread plating anaerobically at 37±2°C for 24–48 h. Two different media were used for isolation: DSMZ Medium 869 and ATCC Medium 1365 purged with 100% N_2_ were used for the isolation of rumen *Streptococcus* species.

### Morphological, physiological, and biochemical analysis

Microbial colonies were picked and transferred separately to 50 mL screw cap bottles containing reinforced clostridial broth (RCB) medium (pre-sterilized and purged with O_2_–free nitrogen). Reinforced clostridial medium (RCM) is one of the synthetic media that favor the growth of anaerobic bacteria used herein. The bottles were incubated at 37±2°C for 24–48 h under static conditions. The pure colonies grown on RCM were analyzed for physiological, morphological, and biochemical characteristics. The morphological properties of the colonies including size, shape, color, margin, elevation, opacity, and consistency, were evaluated. Gram staining and spore staining were performed using Gram staining kit (K001) and Schaeffer and Fulton’s Spore Stain-Kit (K006). Pure colonies were streaked onto blood agar medium and incubated at 37±2°C for 72–96 h to study hemolysis mechanism. In addition, the strains were studied for tannin degradation and catalase production as per the methods described by Tahmourespour *et al*. [12] and Dekker and Lau [13], respectively.

### 16S rRNA gene sequencing and phylogenetic analysis

Two distinctly different pure cultures were selected for microbial identification studies. They were inoculated into 50 mL of RCB medium and incubated at 37±2°C for 24 h. The cell broth was then diluted 10-fold and subjected to a heat treatment to lyse the cells (95°C for 5 min). It was followed by genomic DNA extraction of the strains using Ezup Column Bacterial Genomic DNA Purification Kit. The 16S rRNA gene was targeted for microbial identification using 27F 5’ GAGTTTGATCMTGGCTCAG 3’ and 1492R 5’ TACGGYTACCTTGTTACGACTT 3’ eubacterial primers. The amplification was performed using the polymerase chain reaction (PCR) program: 94°C for 30 s, 55°C for 1 min, 72°C for 1 min, 35 cycles, and 72°C for 5 min. The PCR product was purified with a SanPrep column DNA gel extraction kit and sequenced by a DNA analyzer (3730×l, Applied Biosystems). The resulting sequences were subject to the National Center for Biotechnology Information (NCBI) basic local alignment search tool (BLAST) analysis against other bacterial 16S rRNA gene sequences from the GenBank database. Sequence alignment and phylogenetic analysis were performed by MEGA6 (https://www.megasoftware.net/) with a bootstrap method. The evolutionary distances were analyzed by the maximum composite likelihood method and were shown as the number of base substitutions per site.

### Powder formulation of *S. bovis*

Isolated *S. bovis* was used in the RUSITEC challenge experiment as a model microorganism. A vial of glycerol stock of *S. bovis* was inoculated into 100 mL of RCB medium followed by incubation at 37±2°C at 150 rpm for 120 h. The optical density of cell biomass was recorded at 24 h intervals up to 120 h, using a ultraviolet (UV)–visible spectrophotometer (Thermo Fisher Scientific, US) at 600_nm_, to determine the logarithmic growth phase of *S. bovis*. A pre-seed of 1% was inoculated into 1000 mL of RCB medium, followed by incubation at 37±2°C, at 150 rpm, until the logarithmic growth phase. This cell broth, termed as the seed culture, was then diluted serially using pre-sterilized 0.85% saline, to define the colony-forming units (CFU)/mL. The seed culture of *S. bovis* was centrifuged at 4000×g for 30 min, and the obtained pellet was resuspended in 10 mL mixture of tween 80 (0.1 g/L)+saline (9 g/L), corresponding to 5 mL volume followed by 5 mL of 50% glycerol. All solutions were subjected to three cycles of vacuum (3 min) and N_2_ (1 min) to maintain the anaerobicity. The cell mixture, termed as lyo–slurry was frozen at −80°C for 2 h followed by lyophilization at −55°C for 36–48 h using a Heto PowerDry LL3000 Freeze Dryer. Total viable count (TVC) estimation was performed using 1 mL of cell broth and lyo–slurry and 1 g of lyophilized cell biomass mixed with 0.85% pre-sterilized saline. The mixture was then diluted serially from 10^1^ to 10^10^ and plated over RCM as described above. The TVC of lyo–slurry and lyophilized cell biomass was expressed as CFU/mL and CFU/g, respectively. Further, the dry cell weight (yield) was determined.

### Probiotic formulation (PF)

The PF developed in-house, PF 1, had a bacterial composition of *Bacillus subtilis* (MTCC 2414), *Bacillus amyloliquifaciens* (MTCC 10456), and *Propionibacterium freudenreichii* (NCIM 2111) with CFU/mL of 7×10^6^, 5×10^6^, and 4.8×10^6^, respectively.

### RUSITEC experiment

The RUSITEC (M/s Eaga Tools and Instruments, Chennai, India) apparatus consisted of four cylindrical chambers, treated as independent fermentation vats. In the present study, the four chambers were designated as A−D. The experiment involved a control which had not been exposed to any probiotic or pathogen intervention and simulated the native conditions of an animal rumen. In the beginning, sheep rumen digesta was obtained from a slaughterhouse under controlled environmental conditions. The rumen content was washed and diluted as before. The filtered rumen was used for TVC estimation, representing the d0 population size. The filtered rumen content was further diluted to 4 L using artificial saliva. The RUSITEC operations were conducted for 7 days to generate the data for one experiment.

The experiment was initiated with inoculation of 800 mL of filtered rumen content in each of the four RUSITEC chambers, having 1 L capacity. Crude rumen solids (80 g) were suspended in each RUSITEC chamber, each with a nylon bag of 100 µm pore size. Chamber A was designated as the control, whereas B–D chambers were challenged with probiotics or pathogens. The assembly was fitted into the water bath maintained at 38±2°C temperature. The rumen content inside the RUSITEC chambers was stirred continuously at 20 rpm. Artificial saliva was infused in a continuous manner at a rate of 0.20 mL/min. Saliva feeding was initiated after 6 h of stabilization.

After 24 h, nylon bags were removed from the feed vessel. Every morning, mixed cattle feed (Table-1) was provided for chambers A–D at a dosage of 1 g/d, replacing the rumen solids in the nylon bags. Chamber B–D were supplemented with 0.1 g PF 1, *S. bovis*, and PF 1+*S. bovis*, respectively. The digested fluid was collected in 1000 mL Borosil bottles, attached to the RUSITEC chambers. Fermentation gas was collected in gas bags attached to effluent collection bottles. The feed digestibility was calculated on the basis of compositional analysis of d0 and d7 residual feed as per the method of Neubert *et al*. [14]. Fermented RUSITEC fluid from control and test chambers was collected for TVC estimation until d7. Three RUSITEC replications were conducted to determine the results of probiotic or pathogen performance *in vitro*.

**Table 1:** Ingredient and chemical components of diet.

Nutrient components	Quantity (% w/w)
Moisture content	70.31
Solids	29.69
Ash	3.23
Fiber content	37.42
Total kjeldahl nitrogen	13.48
Carbohydrate and lignin	
Glucose	28.98
Xylose	14.33
Arabinose	2.15
Acid insoluble lignin	14.91

### Volatile fatty acids (VFAs), lactic acid, and pH analysis

The effluent collected per day (60 mL) was used for pH and VFA assessment. These samples were collected daily before introducing a new feed and analyzed for ruminal VFA concentration (acetic, propionic and butyric acids, isobutyric acid, isovaleric acid, and valeric acid, mmol/L) from d0 to d7 using gas chromatography (Agilent, 7890A series). The lactic acid concentration was determined using high-performance liquid chromatography (Agilent 1200 series).

### TVC estimation

The original rumen sample of d0 and a fermented rumen sample of d7 were selected for TVC estimation of total strict anaerobic bacteria, coliforms, aerobic bacteria, yeasts, and facultative anaerobic bacteria. The procedure used the following media: RCM, MacConkey’s agar, Nutrient agar, Yeast extract Peptone Dextrose agar, and De Man, Rogosa, and Sharpe agar, respectively.

For estimation, 1 mL of sample was serially diluted (10–fold increments) using tween 80 (0.1 g/L)+saline (9 g/L), followed by spread plating of 100 µL of each dilution on respective medium. Plates were incubated at 37±2°C for 48 h under suitable conditions of anaerobicity, and TVC was determined.

### Bacteriocin-like inhibitory substance (BLIS) activity

*S. bovis* was streaked onto RCM plates and incubated at 37±2°C for 24 h. A single isolated colony was inoculated into fresh RCB and incubated at 37±2°C for 24–48 h under static conditions. The cell broth was centrifuged at 4000×g for 10 min. The resulting supernatant was filtered through a 0.2 μm filter (Acrodisc^®^ Syringe Filters, Pall), and further concentrated to 5×concentration using rotary evaporator (Heidolph Hei-VAP Advantage). The resulting concentrate was subjected to agar diffusion assays as per the method described by Zhang *et al*. [15].

The indicator bacteria were *Escherichia coli* (NCIM 2931) and *Salmonella enterica* (ATCC 13311). They were inoculated into the nutrient broth and incubated at 37±2°C for 24 h. The optical density of the culture was determined at OD_600_ using a UV–visible spectrophotometer (Thermo Fisher Scientific, US).

Nutrient agar plates with 5 mm diameter wells were used for the agar diffusion assay. For inhibition testing, 100 µL of an indicator strain at an OD_600_ of 0.09–0.1 was spread plated. Cell-free extract (CFE) (100 µL) was pipetted into each well. Streptomycin (100 mg/L, Sigma Aldrich, USA) and 100 µL nutrient broth were used as positive and negative controls, respectively. The assay plates were pre-incubated at 4°C for 2 h to increase the compound diffusion across the medium. Further incubation was conducted at 37±2°C for 48 h to determine the antimicrobial activity.

### Statistical analysis

The significant differences between acetic acid, propionic acid, and butyric acid profiles were analyzed between control, PF 1, *S. bovis*, and PF 1+*S. bovis* intervention samples using multivariate one-way analysis of variance model (MANOVA), where the response of VFA was determined against the independent variables of probiotic, pathogen, or mixed interventions. Similarly, significant differences between the TVC of different types of bacteria were estimated between control, PF 1, *S. bovis*, and PF 1+*S. bovis* intervention samples using MANOVA. Differences between means were considered significant at p=0.05. Statistical analysis was conducted using Minitab^®^ 17.1.0 (Minitab Inc.).

## Results

### Isolation and characterization of rumen *Streptococcus species*

**Figure-1:**
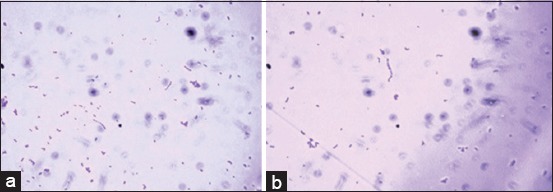
Gram staining of (a) Strain 1 and (b) Strain 2 as Gram-positive cocci; single, diplococci or in chain of 4-10 cells.

**Figure-2:**
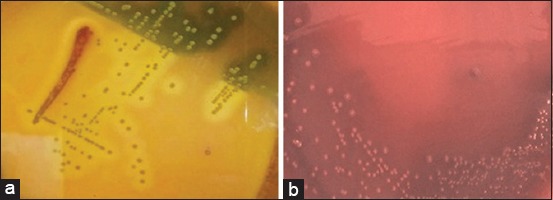
Hemolytic activity of rumen *Streptococcus* spp. (a) Beta hemolysis of Strain 1 within 18 h, (b) alpha hemolysis of Strain 2 within 18-96 h.

Among 15 distinct colonies that were obtained from unfiltered and filtered sheep rumen contents, two pure colonies, defined as Strain 1 and Strain 2, exhibited microscopic resemblance to genus *Streptococcus*, appearing as Gram-positive cocci that were single, diplococci, or in chains of 4-10 cells (Figure-1a and b). The pure colonies were examined for morphological properties including size, shape, color, margin, elevation, opacity, and consistency on selective isolation medium, RCM, and blood agar medium, as shown in Table-2. Spore staining indicated the strains to be non-spore formers. Hemolysis determination indicated beta-and alpha-hemolysis for Strain 1 and Strain 2 within 18 h and 18-96 h, respectively (Figure-2a and b). The strains could not degrade tannin and were catalase-negative.

**Table 2:** Colony characteristics of rumen *Streptococcus* species.

Strains	Medium	Size (mm)	Shape	Color	Margin	Elevation	Opacity	Consistency
Strain 1	Medium 1365 (ATCC)	1	Circular	Pink	Irregular	Flat	Opaque	Smooth
	Reinforced clostridial agar	1	Circular	White	Entire	Flat	Transparent	Moist
	Blood agar	1	Circular	White	Entire	Flat	Opaque	Smooth
Strain 2	Medium 869 (DSMZ)	>1	Circular	Yellowish	Entire	Flat	Opaque	Moist
	Reinforced clostridial agar	2	Circular	White	Entire	Raised	Opaque	Moist
	Blood agar	1	Circular	Yellowish	Entire	Flat	Opaque	Smooth

Strain 1 grew pink colonies on ATCC Medium 1365 (Table-2). It is noteworthy that the pigmented *Streptococcus* strains grew on a non-recommended ATCC Medium 1365.

The gram nature and hemolysis property of strains were observed to be similar to ruminal *Streptococcus* species as reported by Spellberg and Brandt [16].

### 16S rRNA gene sequencing and identification

**Figure-3:**
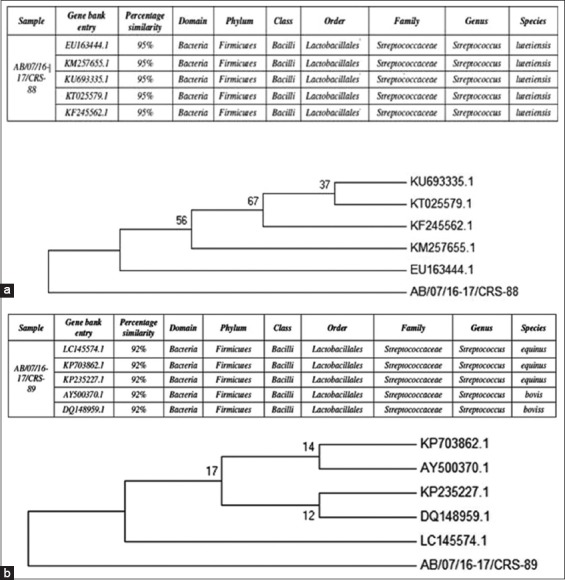
Phylogenetic analysis of (a) *Streptococcus lutetiensis* (Strain 1) and (b) *Streptococcus bovis* (Strain 2). Sequence alignment and phylogenetic analysis were performed by MEGA6 with a bootstrap method. The evolutionary distances were analyzed using the maximum composite likelihood method.

The 16S rRNA gene sequencing and identification confirmed the strain identities to be *Streptococcus* *lutetiensis* (Strain 1) and *S. bovis* (Strain 2), respectively (Figure-3a and b). In the present research, Strain 1 and Strain 2 were found to share 95% and 92% sequence similarity with *S. lutetiensis* and *S. bovis*, respectively.

### Powder formulation of *S. bovis*

The optical density of *S. bovis* biomass gradually increased from 0.2±0.05 to 2.5±0.2 within 24 to 72 h, after which it declined to 1.2±0.2 by 120 h. Log phase cell culture of *S. bovis* had 9×10^10^ CFU/mL and had cell biomass of 1 g/L. The cell count of the final formulation for testing was 7×10^8^ CFU/mL.

### VFAs, lactic acid, and pH analysis

**Figure-4:**
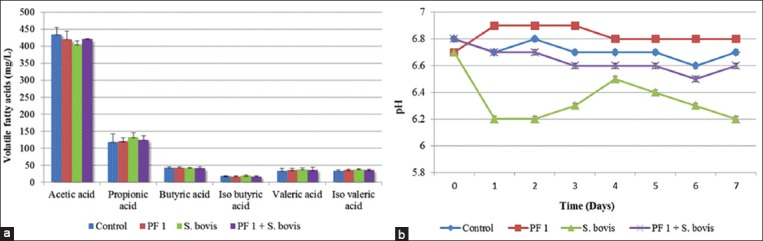
Fermentation profile of *in vitro* rumen simulation technique studies including (a) volatile fatty acid profile and (b) pH profile of control, probiotic formulation (PF) 1, *Streptococcus bovis* and PF 1+*S. bovis* from day 1 to day 7.

The effect of administration of *S. bovis* was evaluated using RUSITEC system and compared with in-house developed probiotic, PF 1. Results of chemical analysis, including VFA, lactic acid, and pH profiling, are shown in Figure-4a and b.

Among the VFA analyzed, there was reduction of 5% (405±10 mg/L) in acetic acid concentration with *S. bovis* intervention in comparison with control (435±20 mg/L), PF 1 (420±25 mg/L), and PF 1+*S. bovis* (421±12 mg/L). *S. bovis* dosage led to a linear rise in the propionic acid concentration (132±15 mg/L) as compared to control (118±25 mg/L). There was a significant difference (p<0.05) between acetic acid and propionic acid levels after intervention with *S. bovis*. No major change in butyric acid, isobutyric acid, or isovaleric acid concentrations were observed in any RUSITEC chamber. Administration of *S. bovis* resulted in an increase in valeric acid concentration to 38±5 mg/L as compared to control (34±7 mg/L), PF 1 (36±5 mg/L), and PF 1+ *S. bovis* (36±9 mg/L) dosage, respectively. There was no significant change in the pH in any of the chambers except for the chamber with *S. bovis* intervention, where it decreased from 6.8±0.02 to 6.2±0.01. Lactic acid was not detected as a major byproduct and was in the range of 0.02±0.001% w/w.

### TVC estimation

**Figure-5:**
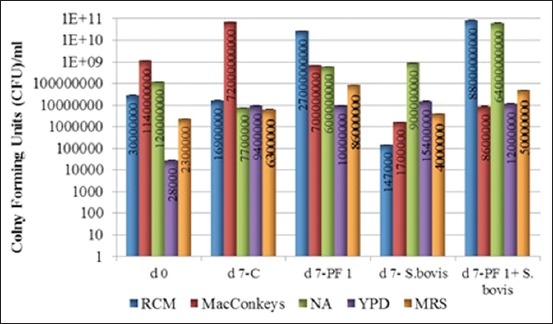
Day 0 versus day 7 total viable count estimation of strict anaerobic bacteria, coliforms, aerobic bacteria, yeast and facultative anaerobic bacteria for probiotic formulation (PF) 1, *Streptococcus bovis* and PF 1+*S. bovis* interventions in comparison with control.

Seven days of RUSITEC operations with *S. bovis*, PF 1, PF 1+*S. bovis* administrations revealed a distinct pattern of TVC from each specific medium used, as shown in Figure-5.

The d0 TVC of strictly anaerobic bacteria estimated to be 3.0×10^7^ CFU/mL declined to 1.69×10^7^ CFU/mL under control conditions of d7. PF 1 and PF 1+*S. bovis* interventions increased the same to 2.7×10^10^ CFU/mL and 8.8×10^9^ CFU/mL, respectively. However, administration of *S. bovis* led to a highly reduced count of 1.47×10^5^ CFU/mL, corresponding to a 30.89% decrease. In a conclusive way, d7 TVC of strictly anaerobic bacteria was induced by PF 1 (39.51%) and PF 1+*S. bovis* (46.37%) dosage as compared to d0 TVC.

Coliforms, a type of detrimental bacteria for ruminants, leading to mastitis like conditions, had d0 count of 1.14×10^9^ CFU/mL, which was observed to be 7.2×10^9^ CFU/mL under the control conditions of d7. PF 1 and PF 1+*S. bovis* interventions similarly reduced the coliform TVC to 7.0×10^8^ CFU/mL (2.33%) and 8.60×10^6^ CFU/mL (23.43%), respectively. *S. bovis* administration drastically reduced the coliform population to 1.7×10^6^ CFU/mL (31.20%), indicating the possible production of a bacteriocin-like substance by *S. bovis*.

The d0 count of aerobic bacteria initially estimated to be 1.2×10^8^ CFU/mL decreased to 7.7×10^6^ CFU/mL under control conditions of d7. PF 1 and PF 1+*S. bovis* interventions lead to a linear rise to 6×10^8^ CFU/mL (8.65%) and 6.4×10^10^ CFU/mL (33.75%), respectively. Independent dosage of *S. bovis* increased the value to 9×10^8^ CFU/mL (10.83%).

The d0 yeast population was recorded to be 2.8×10^4^ CFU/mL and was increased to 9.4×10^6^ CFU/mL under control conditions at d7. PF 1 and PF 1+*S. bovis* interventions raised the value to 1×10^7^ CFU/mL (57.40 %) and 1.2×10^7^ CFU/mL (59.18%), respectively. There was a significant rise in yeast TVC with *S. bovis* administration, at 1.54×10^7^ CFU/mL (61.62%).

The facultative anaerobic bacteria had TVC of 2.3×10^6^ CFU/mL on d0. Under control conditions, by d7, there were 6.3×10^6^ CFU/mL. PF 1 and PF 1+*S. bovis* administrations lead to a log increase to 8.6×10^7^ CFU/mL (24.72%) and 5×10^7^ CFU/mL (21.02%) in comparison with an independent dosage of *S. bovis*, which had a value of 4×10^6^ CFU/mL (3.77%).

There was a significant difference (p<0.05) between the TVC of strict anaerobic bacteria, coliforms, aerobic bacteria, yeasts, and facultative anaerobic bacteria, depending on the intervention with probiotics or pathogens or their combinations thereof.

### BLIS activity

**Figure-6:**
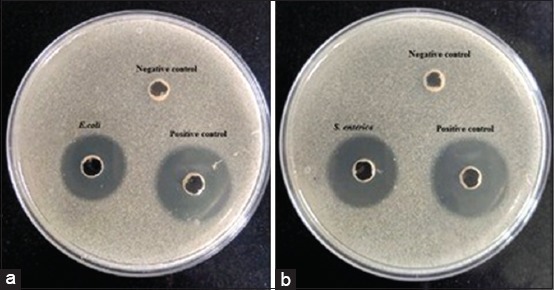
Antimicrobial activity testing of 5×concentrated cell-free extract of *S. bovis* against (a) *Escherichia coli* and (b) *Salmonella enterica* using agar diffusion assay. Streptomycin and nutrient broth have been used as positive and negative control, respectively.

The 5× concentrated CFE of *S. bovis* exhibited strong antibacterial activity against *E. coli* (NCIM 2931) and *S. enterica* (ATCC 13311), as represented in Figure-6a and b. The 5×concentrated CFE of *S. bovis* displayed a 12 and 14 mm zone of inhibition against *E. coli* and *S. enterica*, respectively, justifying further research on bacteriocin and the inhibition spectrum.

## Discussion

The microbial community inhabiting the rumen is diverse. The microbes live in a symbiotic relationship and functionally interact with the host, playing an imperative role in maintaining a stable intraruminal environment and bacterial ecosystem. In ruminants fed conventional diets, the type of carbohydrate consumed modifies the rumen microbial population [17]. The fermentation of high grain diets with starch and sugars digested by amylolytic bacteria leads to the production of pyruvic acid, and subsequently VFA, causing a drop in ruminal pH. This drop causes many pH-sensitive Gram-negative bacteria to decrease, including lactic acid-consuming bacteria, such as *M. elsdenii* and *S. ruminantium*. Conversely, the lactic acid-producing Gram-positive bacteria, especially the *S. bovis* population, increases leading to ruminal acidosis [18].

Ruminal acidosis is a bovine disease, which affects feedlot and dairy cattle. By definition, acidosis is a decrease in the alkali in body fluids relative to the acid content [19]. It leads to irregularity in feed intake, poor digestibility, reduced milk yield and quality, damage to gastrointestinal tract, liver lameness, and abscesses [20]. In this scenario, bacterial probiotics are known to provide positive post-rumen effects for the animal by improving the population of beneficial gut microflora, by altering rumen fermentation to reduce the risk of ruminal acidosis [21].

In this context, a combination of *Lactobacillus* and *Enterococcus* probiotics is known to improve ruminal performance [22]. The mechanism of lactic acid bacteria (LAB) based probiotics is not yet clear, but their administration is thought to help the rumen microflora adapt to the presence of lactic acid, and thereby prevents lactic acid accumulation in the rumen [23].

Previous research has been targeted towards the analysis of the administration of *S. bovis* on fermentation characteristics and nutritive value of Tanzania grass silage [8] and of the administration of wheat bran+rumen-isolated *S. bovis* on effective degradation of guinea pig silage [9]. The present study explores the effect of the administration of ruminal *S. bovis* on fermentation characteristics and microbial population in RUSITEC chambers fed with mixed cattle feed. This has been assessed in comparison to a control, in-house developed probiotic PF 1, with a combination of PF 1+*S. bovis*, and only *S. bovis*.

There are numerous reports on isolation and characterization of *Streptococcus* species from the rumen. Researchers have used different media, such as nutrient agar, yeast starch agar, modified membrane-bovis agar, and some specific media incorporated with mineral solution, resazurin, hemin, and cellulose [24]. The current study uses semi-synthetic Medium 1365 ATCC and Medium 869 DSMZ, based on the literature. Furthermore, RCM was assessed for its suitability to revival and maintain original anaerobic bacterial strains of the rumen due to its simplicity, synthetic nature, and suitability for anaerobic bacteria. The present study led to the isolation of genus *Streptococcus*, including *S. lutetiensis* and *S. bovis*. Medium 1365 (ATCC) favored an isolation of *Streptococcus* species, despite not being recommended as a selective medium. Our results indicate that *Streptococcus* species, belonging to one of the dominant groups of lactic acid-producing bacteria, may grow on non-selective media under suitable environmental conditions, thus recommending exploration of various media for isolation of ruminal *Streptococcus* species. The isolated strains matched with previous strains identified by Spellberg and Brandt [16], in terms of Gram staining, spore-forming nature, and catalase activity. This was also confirmed by 16S rRNA gene sequencing and identification. Phylogenetic analysis using a bootstrap method revealed 95% and 92% sequence similarity of Strain 1 and Strain 2 to *S. lutetiensis* and *S. bovis*, respectively.

Group B streptococci are known for pigment production on some distinct media [25]. The present study describes the rumen isolated strains *S. lutetiensis* and *S. bovis*, which belong to Lancefield Group D Streptococci. The isolated *S. lutetiensis* showed pink pigmentation on ATCC Medium 1365. This is an indication of possible pigment production by Group D streptococci which has not previously been reported. Furthermore, the present study detected alpha hemolysis by *S. bovis* as similar to the previous research by Spellberg and Brandt [16].

To the best of our knowledge, this is the first report wherein rumen-isolated *S. bovis* used in RUSITEC under mixed cattle feed diet caused changes in rumen fermentation and microbial population. We observed that administration of *S. bovis* under mixed cattle feed diet increased the propionate levels, which is in agreement with Meissner *et al*. [26] who stated that there could be upregulation of *Propionibacteria* under lactic acid overproduction. In general, *Propionibacteria* are common inhabitants in the rumen but are typically present in low numbers [27]. In support of this, the current study reported an increase in the total yeast population by 61% with *S. bovis* administration in RUSITEC. Miller-Webster *et al*. [28] reported a reduction in the molar proportion of acetic acid and an increase in propionic acid with yeast administration. The rise in yeast population between the PF 1 intervention (57.40%) further increased to 59.18% with PF 1+*S. bovis* administration, indicating the stimulatory effect of *S. bovis* for the yeast population. This was also confirmed by independent administration results of *S. bovis* explained earlier. *S. bovis* normally accounts for <1% of the ruminal bacteria (approximately 10^7^ cells/mL ruminal fluid), but it can dominate the population when soluble carbohydrates (e.g., starch or sugars) are plentiful. Homolactic fermentation produces very little adenosine triphosphate (ATP) per hexose, but *S. bovis* has a very fast rate of fermentation and can generate more ATP per hour than other ruminal bacteria [29]. This can lead to sub-acute rumen acidosis under starch-rich diet where ruminal pH stays in the range of 5.2–6 for a prolonged period [30]. In the current study, we reported a slight decrease in pH from 6.8±0.02 to 6.2±0.01 in the vessel with *S. bovis* from d1 to d7. In our study, mixed cattle feed diet prevented the domination of *S. bovis* that allowed monitoring of its behavioral pattern.

Fouladgar *et al*. [31] reported a reduction in fecal coliforms in veal calves after administration of *Lactobacillus* spp. probiotic. We observed that there was a 19.87% rise in the coliform population on d7 under control conditions of RUSITEC. However, probiotic PF 1 led to a coliform reduction of 2.33%. Furthermore, we reported the highest reduced of 31.20%, under *S. bovis* intervention. Bacteriocins produced by LAB have been reported for their ability to permeate the outer membrane of Gram-negative bacteria and subsequently induce the inactivation of Gram-negative bacteria. The effects are enhanced in conjunction with other enhancing antimicrobial environmental factors such as low temperatures, organic acids, and detergents [32]. The combined action of PF 1+*S. bovis* resulted in a 23.40% coliform reduction clarifying the parallel and enhanced coliform reduction as compared to that of the only intervention of PF 1.

It has also been reported that 20% of rumen isolated *Streptococcus* species and 50% of ruminal *S. bovis* strains produce BLIS, displaying a wide range of specificity and potency [10]. Wang *et al*. [33], Azevedo *et al*. [34], and Xiao *et al*. [35] characterized bovicin HJ50 such as lantibiotic, bovicin HC5, and bovicin H550, respectively, in earlier studies. In the present study, the *S. bovis*-mediated reduction in coliform population was elucidated further by examining its extracellular antimicrobial activity using 5×concentrated CFE against representative coliforms including *E. coli* and *S. enterica*. The positive response in terms of zone of inhibition implicated the possible production of a bacteriocin, bovicin, which is worthy of further study.

Similarly, the total anaerobic bacteria were reduced by 3.33% on d7 under control conditions of RUSITEC but increased under PF 1 intervention to 39.51%. In the current study, probiotic PF 1 may have upregulated predominant phyla *Bacteroides*, *Firmicutes*, and *Synergistetes*, resulting in improved cellulolysis and rumen functions [36]. On the other hand, *S. bovis* administration reduced the total anaerobic bacteria by 30.89%. This may impact cellulose degradation efficiency of the rumen. However, a 46.37% increase was reported under mixed supplementation of PF 1+*S. bovis*. This indicates that *S. bovis* may not function similarly when administered individually or in conjunction with other probiotic microbes.

The total facultative anaerobic bacteria and total aerobic bacteria showed a rise of 6.87% and decrease of 14.76%, respectively, on d7 under control conditions. These counts were upregulated under all interventions of PF1, *S. bovis*, and PF 1+*S. bovis*. Although the PF 1 intervention triggered aerobic bacterial population, the highest rise in aerobic bacteria was observed with PF 1+*S. bovis* administration, followed by *S. bovis* intervention, respectively, indicating the causal effect of acidosis.

## Conclusion

Novel strains of *S. lutetiensis* and *S. bovis* were isolated from sheep rumen digesta. The 16S rRNA gene sequencing and identification followed by NCBI BLAST revealed 95% and 92% sequence similarity to reference *S. lutetiensis* and *S. bovis*. Beta and alpha hemolysis by *S. lutetiensis* and *S. bovis* indicates the differing characters of *S. bovis* group biotypes. To the best of our knowledge, for the first time, we report pink pigmentation by *S. lutetiensis* on Medium 1365 ATCC. Supplementation of *S. bovis* in the rumen and the subsequent physiological, microbiological, and functional changes led to the understanding that *S. bovis* is a rapid colonizer of the rumen. It exhibits the principle of exclusivity by reducing the count of coliform microbes. A substantially increased count of yeast indicates the symbiosis between the two, which should be investigated further. Further such studies would indicate the role and microbial associations in the complex rumen, leading to better design of interventional studies and products.

## Authors’ Contributions

DA and AK designed the research. DA collected the samples, performed the research, analyzed data, and prepared the manuscript. AK guided the entire research, data analysis, and manuscript preparation. DA and AN performed *S. bovis* lyophilization. DA and SL performed RUSITEC operations and data analysis. All authors have read and approved the final manuscript.

## References

[1] Weimer P.J (2015). Redundancy, resilience, and host specificity of the ruminal microbiota:implications for engineering improved ruminal fermentations. Front. Microbiol.

[2] Jans C, Meile L, Lacroix C, Stevens M.J.A (2015). Genomics, evolution, and molecular epidemiology of the *Streptococcus bovis/Streptococcus equinus* complex (SBSEC). Infect. Genet. Evol.

[3] Wang H, Pan X, Wang C, Wang M, Yu L (2015). Effects of different dietary concentrate to forage ratio and thiamine supplementation on the rumen fermentation and ruminal bacterial community in dairy cows. Anim. Prod. Sci.

[4] Chen L, Liu S, Wang H, Wang M, Li Y (2016). Relative significances of pH and substrate starch level to roles of *Streptococcus bovis*S1 in rumen acidosis. AMB Express.

[5] Granja-Salcedo Y.T, Junior C.S.R, de Jesus R.B, Gomez-Insuasti A.S, Rivera A.R, Messana J.D, Canesin R.C, Berchielli T.T (2016). Effect of different levels of concentrate on ruminal microorganisms and rumen fermentation in Nellore steers. Arch. Anim. Nutr.

[6] Wetzels S.U, Eger M, Burmester M (2018). The application of rumen simulation technique (RUSITEC) for studying dynamics of the bacterial community and metabolome in rumen fluid and the effects of a challenge with *Clostridium perfringens*. PLoS One.

[7] Ghali M.B, Scott P.T, Alhadrami G.T, Al Jassim R.A.M (2011). Identification and characterization of the predominant lactic acid-producing and lactic acid-utilizing bacteria in the foregut of the feral camel (*Camelus dromedarius*) in Australia. Anim. Prod. Sci.

[8] Zanine A.M, Bonelli E.A, de Souza A.L, Ferreira D.J, Santos E.M, Ribeiro M.D, Geron L.J, Martins R, Pinho A (2016). Effects of *Streptococcus bovis* isolated from bovine rumen on the fermentation characteristics and nutritive value of Tanzania grass silage. Sci. World. J.

[9] Bonelli E.A, Zanine A.M, de Souza A.L, Ferreira D.J, Alves G.R (2013). Ruminal degradability of guinea grass silage inoculated with *Streptoccocus bovis* isolated from bovine rumen combined or not with com wheat bran. J. Agric. Sci.

[10] Joachimsthal E.L, Reeves R.K.H, Hung J, Nielsen L.K, Ouwerkerk D, Klieve A.V, Vickers C.E (2010). Production of bacteriocins by *Streptococcus bovis* strains from Australian ruminants. J. Appl. Microbiol.

[11] Pers-Kamczyc E, Zmora P, Cieslak A, Szumacher-Strabel M (2011). Development of nucleic acid-based techniques and possibilities of their application to rumen microbial ecology research. J. Anim. Feed Sci.

[12] Tahmourespour A, Tabatabaee N, Khalkhali H, Amini I (2016). Tannic acid degradation by *Klebsiella* strains isolated from goat feces. Iran. J. Microbiol.

[13] Dekker J.P, Lau A.F (2016). An update on the *Streptococcus bovis* group:Classification, identification, and disease associations. J. Clin. Microbiol.

[14] Neubert A.M, Vanamburgh F, John J.L (1940). Determination of crude fiber. Ind. Eng. Chem. Anal. Ed.

[15] Zhang H, Cui Y, Zhu S, Feng F, Zheng X (2010). Characterization and antimicrobial activity of a pharmaceutical microemulsion. Int. J. Pharm.

[16] Spellberg B.A, Brandt C, Jorgensen J.H, Pfaller M.A, Carroll K.C, Funke G, Landry M.L, Richter S.S, Warnock D.C (2015). Streptococcus. Manual of Clinical Microbiology.

[17] AlZahal O, Li F, Guan L.L, Walker N.D, McBride B.W (2016). Factors influencing ruminal bacterial community diversity and composition and microbial fibrolytic enzyme abundance in lactating dairy cows with a focus on the role of active dry yeast. J. Dairy Sci.

[18] Hernandez J, Benedito J.L, Abuelo A, andCastillo C (2014). Ruminal acidosis in feedlot:From etiology to prevention. Sci. World. J.

[19] Dehkordi A.J, Dehkordi Z.K (2011). Occurrence of metabolic alkalosis in rumen lactic acidosis:A review article. Comp. Clin. Pathol.

[20] Abdela N (2016). Sub-acute ruminal acidosis (SARA) and its consequence in dairy cattle:A review of past and recent research at global prospective. Achiev. Life Sci.

[21] Belanche A, Doreau M, Edwards J.E, Moorby J.M, Pinloche E, Newbold C.J (2012). Shifts in the rumen microbiota due to the type of carbohydrate and level of protein ingested by dairy cattle are associated with changes in rumen fermentation. J. Nutr.

[22] Ellis J.J, Bannink A, Hindrichsen I.K, Kinley R.D, Milora N, Bannink A, Dijkstra J (2016). The effect of lactic acid bacteria included as a probiotic or silage inoculant on *in vitro* rumen digestibility, total gas and methane production. Anim. Feed Sci. Technol.

[23] Qadis A.Q, Goya S, Ikuta K, Yatsu M, Kimura A, Nakanishi S, Sato S (2014). Effects of a bacteria-based probiotic on ruminal pH, volatile fatty acids and bacterial flora of Holstein calves. J. Vet. Med. Sci.

[24] Das K.C, Qin W (2012). Isolation and characterization of superior rumen bacteria of cattle (*Bos taurus*) and potential application in animal feedstuff. Open J. Anim. Sci.

[25] Rosa-Fraile M, Dramsi S, Spellerberg B (2014). Group B Streptococcal hemolysin and pigment, a tale of twins. FEMS Microbiol. Rev.

[26] Meissner H.H, Henning P.H, Horn C.H, Leeuw K.J, Hagg F.M, Fouche G (2010). Ruminal acidosis:A review with detailed reference to the controlling agent *Megasphaera elsdenii*NCIMB 41125. Afr. J. Anim. Sci.

[27] Sato S (2016). Pathophysiological evaluation of subacute ruminal acidosis (SARA) by continuous ruminal pH monitoring. Anim. Sci. J.

[28] Miller-Webster T, Hoover W.H, Holt M, Nocek J.E (2014). Influence of yeast culture on ruminal microbial metabolism in continuous culture. J. Dairy Sci.

[29] Hackmann T.J, Firkins J.L (2015). Maximizing efficiency of rumen microbial protein production. Front. Microbiol.

[30] Li S, Danscher A.M, Plaizier J.C (2013). Sub-acute ruminal acidosis (SARA) in dairy cattle:new developments in diagnostic aspects and feeding management. Can. J. Anim. Sci.

[31] Fouladgar S, Shahraki A.D, Ghalamkari G.R, Khani M, Ahmadi F, Erickson P.S (2016). Performance of Holstein calves fed whole milk with or without kefir. J. Dairy Sci.

[32] McDonald P, Edwards R.A, Greenhalgh J.F.D, Morgan C.A, Sinclair L.A (2010). Animal Nutrition.

[33] Wang J, Ma H, Ge X, Zhang J, Teng K, Sun Z, Zhong J (2014). Bovicin HJ50-Like lantibiotics, a novel subgroup of lantibiotics featured by an indispensable disulfide bridge. PLoS One.

[34] Azevedo A.C, Bento B.P, Ruiz J.C, Queiroz M.V, Mantovani H.C (2015). Draft genome sequence of *Streptococcus equinus (Streptococcus bovis*) HC5, a lantibiotic producer from the bovine rumen. Genome Announc.

[35] Xiao H, Chen X, Chen M, Tang S, Zhao X, Huan L (2004). Bovicin HJ50, a novel lantibiotic produced by *Streptococcus bovis*HJ50. Microbiology.

[36] Zhang L.U, Chung J, Jiang Q, Sun R, Zhang J, Zhong Y, Ren N (2017). Characteristics of rumen microorganisms involved in anaerobic degradation of cellulose at various pH values. RSC Adv.

